# Strategies for Enhancing Plant Immunity and Resilience Using Nanomaterials for Sustainable Agriculture

**DOI:** 10.1021/acs.est.4c03522

**Published:** 2024-05-14

**Authors:** Peng Zhang, Yaqi Jiang, Fabienne Schwab, Fazel Abdolahpur Monikh, Renato Grillo, Jason C. White, Zhiling Guo, Iseult Lynch

**Affiliations:** † Department of Environmental Science and Engineering, University of Science and Technology of China, Hefei 230026, China; ‡ Beijing Key Laboratory of Farmland Soil Pollution Prevention and Remediation, College of Resources and Environmental Sciences, China Agricultural University, Beijing 100093, China; § Adolphe Merkle Institute, University of Fribourg, Chemin des Verdiers 4, 1700 Fribourg, Switzerland; ∥ Department of Environmental and Biological Sciences, University of Eastern Finland, Joensuu-Kuopio 80101, Finland; ⊥ Department of Chemical Sciences, University of Padua, Via Marzolo 1, 35131 Padova, Italy; ∇ Department of Physics and Chemistry, School of Engineering, São Paulo State University (UNESP), Ilha Solteira, SP 15385-000, Brazil; ⊗ Department of Analytical Chemistry, The Connecticut Agricultural Experiment Station, New Haven, Connecticut 06504, United States; × School of Geography, Earth and Environmental Sciences, University of Birmingham, Edgbaston, Birmingham B15 2TT, U.K.

**Keywords:** Crop protection agents, biostimulants, nanotechnology, safe-by-design, plant defense, plant science, plant immunity, plant resilience

## Abstract

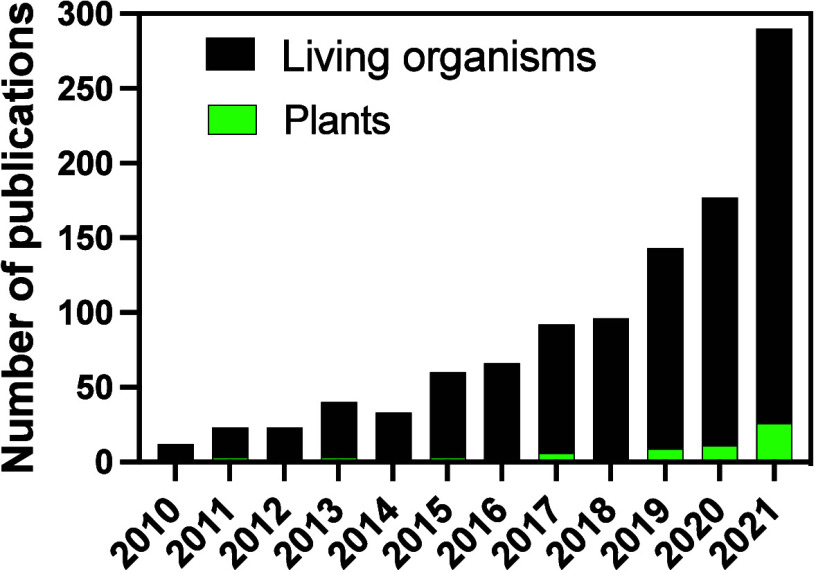

Research on plant-nanomaterial interactions has greatly advanced over the past decade. One particularly fascinating discovery encompasses the immunomodulatory effects in plants. Due to the low doses needed and the comparatively low toxicity of many nanomaterials, nanoenabled immunomodulation is environmentally and economically promising for agriculture. It may reduce environmental costs associated with excessive use of chemical pesticides and fertilizers, which can lead to soil and water pollution. Furthermore, nanoenabled strategies can enhance plant resilience against various biotic and abiotic stresses, contributing to the sustainability of agricultural ecosystems and the reduction of crop losses due to environmental factors. While nanoparticle immunomodulatory effects are relatively well-known in animals, they are still to be understood in plants. Here, we provide our perspective on the general components of the plant’s immune system, including the signaling pathways, networks, and molecules of relevance for plant nanomodulation. We discuss the recent scientific progress in nanoenabled immunomodulation and nanopriming and lay out key avenues to use plant immunomodulation for agriculture. Reactive oxygen species (ROS), the mitogen-activated protein kinase (MAPK) cascade, and the calcium-dependent protein kinase (CDPK or CPK) pathway are of particular interest due to their interconnected function and significance in the response to biotic and abiotic stress. Additionally, we underscore that understanding the plant hormone salicylic acid is vital for nanoenabled applications to induce systemic acquired resistance. It is suggested that a multidisciplinary approach, incorporating environmental impact assessments and focusing on scalability, can expedite the realization of enhanced crop yields through nanotechnology while fostering a healthier environment.

## Abstract 2

Strengthening plants’ defenses without compromising their growth can be achieved by using nanomaterials that induce molecular defense signaling pathways and resistance.

## 1 Introduction

Over the past decade, global food production has come under tremendous pressure as a result of a variety of abiotic and biotic stressors. These stressors include extreme weather, soil salinization, drought, pest and disease outbreaks and other factors.^1^ Meanwhile, research on nanoagrochemicals has greatly advanced over the past decade,^2^ resulting in the first nanoenabled innovations for pest management, plant growth stimulation, and enhanced soil quality.^3−6^ Such discoveries spark hope for a future with more environmentally friendly plant protection solutions, which could be particularly useful in semiarid regions where it is becoming harder to grow food.^7,8^ Here, we provide an overview of the principal concepts to modulate plant immunity using nanotechnology for yield increase.

**Figure 1:**
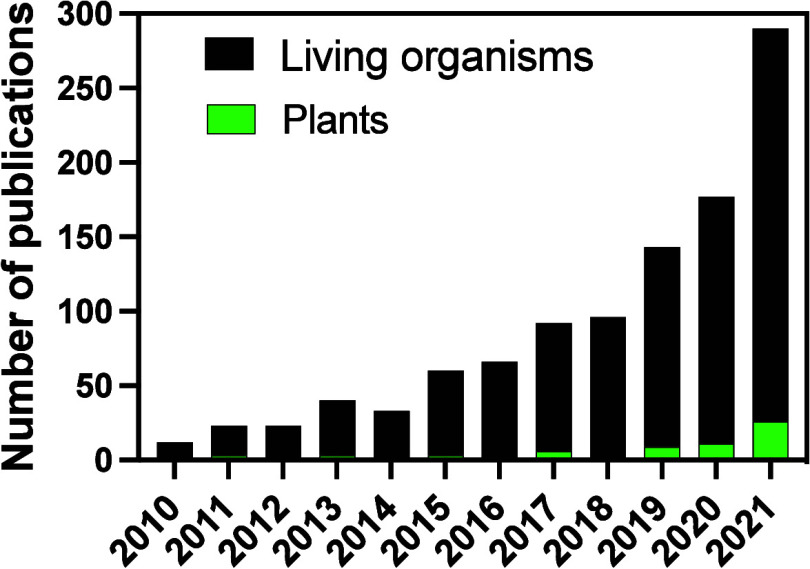
**Publication activity for nano related immunomodulatory effects for plants.** Publication search results using descriptors “nano* ” and “immunomodulatory effects* ” in the ISI Web of Knowledge database from 2010 to 2021. Green data represents articles on plants.

Several nanomaterial classes could be helpful for agricultural applications, including inorganic (e.g., silica(Si), copper(Cu), iron(Fe), zinc(Zn), selenium(Se)), organic (e.g., (bio)polymers such as chitosan, lipids, proteins, and peptides), and hybrid materials.^9^ Laboratory-scale nanoagrochemicals, including pesticides and fertilizers, performed ∼20–30% better than conventional products.^10^ Initially, the primary purpose of nanoagrochemicals was more targeted dosing of active ingredients to plants.^11^ Meanwhile, independent studies have shown that nanoenabled (micro)nutrients such as Si and Cu can surpass the effect expected from the nutrient dose.^5,12^ As a result, the nanomaterials’ immunomodulatory effects have been drawing increased attention from scientists (Figure 1). While immunomodulatory effects are comparatively well-known in animals,^13^ they are still to be understood in plants.

Animals can produce a rapid but weak innate and a slower but more powerful adaptive immune response. T-cell and B-cell lymphocytes (white blood cells) in animals recognize pathogens and cause the production of highly specific antibodies of almost infinite diversity.^14,15^ Nanoimmunomodulation can be designed to target specific immune cells or tissues, thereby precisely modulating the immune response without affecting other cell types. Nanoimmunomodulation can induce immune memory, resulting in quicker and more robust immune responses upon subsequent exposures to the same antigen or pathogen.^16^ In addition, it reduces off-target effects and minimizes adverse effects compared to systemic use of immunomodulatory drugs.^17^ However, the design and optimization of nan-immunomodulation is technically challenging due to the need for precise control of nanoparticle properties. In addition, the development and production of nan-immunomodulation is more costly than traditional immunomodulatory agents or vaccines.

The plants’ immune system works differently. Plants lack the adaptive immune response of a lymph system with circulating defender cells. Nevertheless, using their more developed innate immune system, plants can produce precise immune responses and build a lifelong immunological memory^15^ of many encountered pathogens.^15^ Plants primarily utilize pattern recognition receptors (PRR) to detect conserved microbial molecules known as microbe-associated molecular patterns (MAMP). This mechanism contrasts with that in animals, which employ both pattern recognition receptors and adaptive immune receptors for pathogen detection. Therefore, nanoparticles intended for plant immune modulation may require specific design to effectively interact with these plant-specific PRRs and associated signaling pathways. Additionally, the presence of a rigid cell wall in plant cells, unlike the cellular structure in animals, presents unique challenges for nanoparticle-mediated immunomodulation. Therefore, nanoparticles for use in plants must be able to penetrate or bypass the cell wall to reach intracellular targets or interact with plasma membrane receptors. Surface modification or formulation of nanoparticles may be necessary to enhance uptake or delivery of nanoparticles by plant cells. While approaches for nanoparticle-based immunomodulation in animals can offer valuable insights, they cannot be directly transposed to plant systems due to these fundamental differences in cell structure and immune mechanisms. Tailored strategies are needed in developing nanoimmunomodulatory tools for plants. Investigating and adapting these differences will be crucial in advancing the use of nanotechnology for future agricultural applications, opening new frontiers in enhancing plant immunity and resilience.

**Figure 2:**
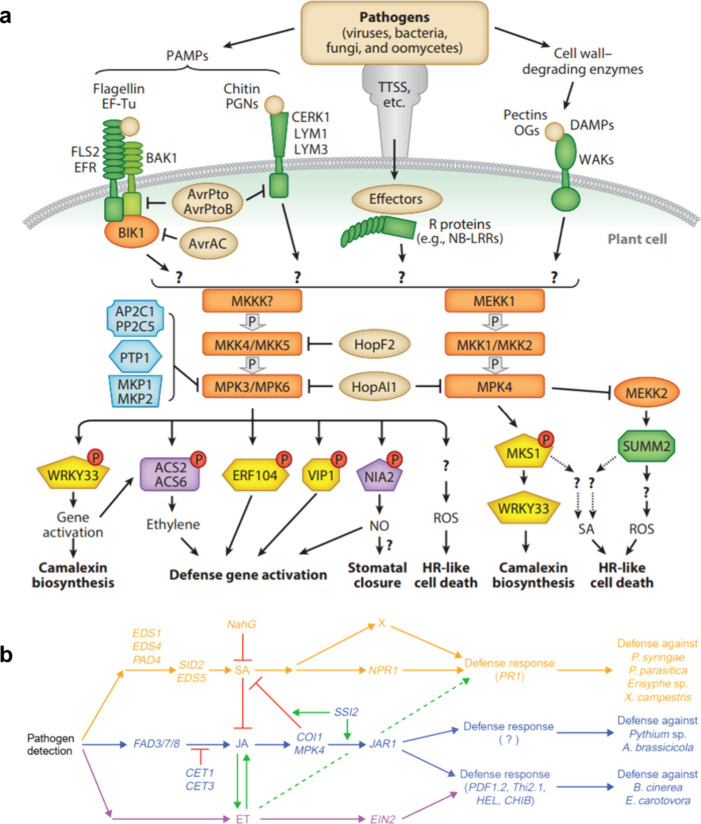
**Key pathogen recognition mechanisms and signaling pathways can be modulated by nanoparticle exposure. a:** Pathogen recognition mechanisms. Figure reproduced from Meng and Zhang 2013.^19^ Pathogens can be recognized via the binding to pattern recognition receptors (PRRs) of a) pathogen-specific molecules - pathogen/microbe-associated molecular patterns (PAMP/MAMP), b) molecules on the surface of the pathogen directly, or c) plant-derived molecules associated with cell damage - damage-associated molecular patterns (DAMP). The ensuing immunity is called PAMP-triggered immunity (PTI). Furthermore, molecules called effectors produced by the pathogen to suppress plant immunity can be recognized by plant-specific resistance proteins (R proteins), which trigger effector-triggered immunity (ETI). After the recognition, different signaling pathways lead to different plant defense responses. For example, mitogen-activated protein kinase (MAPK) cascades (orange) lead to the phosphorylation of target proteins. These, in turn, trigger the signaling by defense hormones such as salicylic acid (SA) and ethylene, the activation of defense genes, the synthesis of antimicrobial metabolites, and reactive oxygen species, including NO. Nanoparticles can most notably interfere with plant immunity before the stage of the MAPK, perhaps by the induction of DAMPs and by the direct generation of reactive oxygen species (ROS).^51^ The signaling components which determine the specific response are an active research field. **b:** Signaling network of three signal transduction pathways of the plant hormones salicylic acid (SA), jasmonic acid (JA), and ethylene (ET). Figure reproduced from Kunkel and Brooks 2002.^33^ Note the mutual antagonistic and synergistic interactions of the SA, JA, and ET pathways.

Plants recognize pathogens or physical damage through complex signaling pathways, which consist of a two-branched surveillance system: pattern-triggered immunity (PTI) and effector-triggered immunity (ETI)^18^ (Figure 2). A pattern, in this context, is a phytopathogen-specific molecule such as bacterial flagellin or fungal chitin (pathogen-associated molecular patterns, PAMPs) or a pathogen damage-associated molecule such as pectic oligosaccharides (damage-associated molecular patterns, DAMPs), that can be detected by pattern-recognition receptors (PRRs, Figure 2) in the plant cell membrane.^15,19^ Pattern-triggered immunity is the first line of a protective reaction in plants, which includes changes in calcium ion (Ca^2+^) influx, ROS, mitogen-activated protein kinase (MAPK) cascade, and the response of defense hormones such as ethylene (ET), abscisic acid (ABA), jasmonic acid (JA), and salicylic acid (SA) (Figure 2).^20^ JA regulates the expression of genes involved in the production of defense compounds and promotes the synthesis of secondary metabolites.^21^ ET regulates the expression of genes involved in cell wall strengthening and activation of defense responses.^22^ ABA also plays a role in plant defense against certain pathogens and interacts with SA and JA pathways to regulate plant stress responses.^23^ If this first PTI fails, a more substantial second line of response, ETI, can be activated.^24^ An effector, in the context of plant immunity, is a pathogen-specific molecule expressed by avirulence (avr) genes, such as *avrRpt2* in *Pseudomonas spp.* bacteria that is released by the pathogen directly into the plant cell to deactivate and circumvent the plant’s first line PTI.^25^ The ETI often results in a hypersensitive response (HR) involving rapid apoptosis-like cell death around the infection site to block the further spread of the pathogen.^14,26^ This vigorous plant response can immunize the whole plant systemically and for life against a broad spectrum of pathogens, a phenomenon called systemic acquired resistance (SAR).^15^

Interestingly, resistance-inducing compounds, organisms, and unspecific abiotic stress can induce a similar, strong, persistent resistance.^27^ Acibenzolar-S-methyl (ASM) is widely reported to induce resistance against a broad spectrum of pathogens in many plant species. For example, ASM reduced the severity of ascomycete blight by 45% to 66% in field trials.^28^ In addition, symbiosis between plants and arbuscular mycorrhizal fungi (AMF) can promote nutrient uptake and thus help plants cope with stress. Previous studies have shown that AMF increased aboveground plant biomass by 87%.^29^ This effect can be used to strengthen plants and is called plant priming, and it also works with nanoparticles (nanopriming).^5,6,30^ Nanopriming involves treating seeds with nanomaterials prior to sowing to improve germination, seedling vigor, and early growth performance. Nanomaterials can be used as seed coatings or incorporated into seed treatments. Initial research on nanopriming has shown that specific nanomaterials elicit the plant immune system, stimulate plant defense responses, and promote plant resistance to stress.^5,6^ For instance, silicon dioxide (SiO_2_) nanoparticles ∼76 nm in diameter can induce systemic resistance acquired in thale cress (*Arabidopsis)* (thale cress, a widely used model plant) in low concentrations by activating salicylic acid, a plant defense hormone promoting SAR.^5^ Along the same line, organic materials such as chitosan nanoparticles ∼90 nm in diameter can increase the levels of defense-related enzymes and activate genes in tea plants (*Camellia sinensis*), a response associated with nitric oxide (NO),^30^ another critical plant defense signaling compound for SAR.^31^ Further, biochar nanoparticles ∼160 nm in diameter can stimulate tobacco plant defense responses and subsequent resistance to the pathogen.^32^ These findings suggest that nanoenabled immunomodulation, or nanopriming, might be an alternative way to enhance plant tolerance, yet the mechanisms are not fully understood.

Here, we discuss the potential mechanisms and environmental impact of nanomaterials’ action on plants’ immune response. We also present our perspective for future work, highlighting several promising strategies to use this knowledge to enhance plant tolerance to biotic and abiotic stress.

## 2 Response to Stress and Defense: Immunity-Related Signaling Pathways with a Possible Role in Nanomaterial Interactions

Different stresses trigger different signaling pathways in plants in crosstalk with each other.^33^ The stress caused by living organisms, such as plant pests and pathogens, is termed biotic stress.^14^ Pressure caused by nonliving environmental factors, such as the climate (heat, cold, flood, drought), pollution (gaseous, aerosols, heavy metals), and high salinity, cause abiotic stress.^34^ As discussed in section 1, biotic stress mainly induces PAMP and effector-triggered immunity (PTI and ETI) in adapted plants (Figure 2a). One crucial signaling pathway in plants susceptible to activated in response to primarily biotic stress is the MAPK cascade (Figure 2b).^19^ The MAPK cascade, which also can be activated by ROS alone,^35^ leads to the phosphorylation of downstream target proteins and ultimately results in the transcriptional regulation of stress-response genes regulating, for example, camalexin biosynthesis, one of many plant-own molecules (phytoalexins) which deter pathogens; stomatal closure to limit pathogen entrance or water loss; cell wall reinforcement; and hypersensitivity reaction and cell death.^19^ The MAPK pathway has been successfully manipulated in animal cells via nanoparticle exposure.^36^ For example, in the field of cancer therapy, nanoparticles are designed to target the MAPK signaling pathway, which plays a key role in cell proliferation, survival and metastasis. Gold nanoparticles functionalized with small interfering RNA (siRNA) targeting components of the MAPK pathway have been shown to inhibit tumors growth and sensitize cancer cells to chemotherapy. By down-regulating MAPK signaling, these nanoparticles can inhibit cancer cell proliferation and promote apoptosis, thereby improving treatment outcomes.^37^ Nanoparticles carrying anti-inflammatory drugs or small molecule inhibitors of MAPK kinases (MEKs) attenuate MAPK activation and reduce the production of pro-inflammatory cytokines in immune cells.^38^ In addition, nanoparticles have been designed to modulate CDPK signaling in immune cells to enhance pathogen clearance and promote host defense mechanisms. Nanoparticle-based delivery of CDPK inhibitors or activators modulates calcium-dependent signaling pathways in macrophages or dendritic cells, resulting in enhanced phagocytosis, cytokine production, and antimicrobial activity against intracellular pathogens. This modulation of CDPK signaling enhances the innate immune response and contributes to the control of infections, thus providing potential therapeutic benefits for infectious diseases.^39^ In plants, although highly promising, little is known about nanoenabled MAPK modulation.

A critical signaling network of plant defense that responds to biotic stress consists of the interconnected signaling pathways of the three plant hormones SA, JA, and ET (Figure 2b).^14,19^ For example, JA and SA are mutually antagonistic, meaning that their specific signaling cascades cannot be activated simultaneously.^33^ This likely ensures the plant uses all its resources for one targeted defense signaling pathway.^33^ Different pathogen species can cause different responses of these hormones, for example, due to distinct herbivore feeding strategies: piercing and sucking pests, such as aphids, primarily trigger SA and JA pathways, whereas the JA pathway predominantly mediates the responses to chewing herbivores.^40^ Recent research has shown that these defense hormones can be directly involved in the reaction of plants to nanoparticle stress.^5,41^

Abiotic stresses like drought, salinity and extreme temperature, does not directly cause an immune response. Nonetheless, abiotic stress can also induce resistance in plants. These stresses may trigger a range of specialized receptors, such as histidine kinase of osmotic stress and ion channels for salinity and heavy metal stress. The signal transduction under abiotic stress might involve a complex network of signaling molecules, including abscisic acid (ABA) for drought, salicylic acid (SA) for heat stress, and ethylene for flooding stress. Nanoparticles interact with plant cells to act as stress sensors or wake-up calls, triggering signals in response to abiotic stresses. These interactions may involve physical interactions with cell membranes, cellular uptake, or regulation of cellular redox state and ion homeostasis. For example, cerium oxide nanoparticles have been shown to enhance drought tolerance in sorghum by altering the expression of ABA-related genes and increasing antioxidant enzyme activities.^42^ Nanoparticles mitigate abiotic stress by scavenging ROS or enhancing antioxidant enzyme activity to reduce oxidative damage; they can modulate stress response to mitigate abiotic stress by regulating the synthesis, signaling, and metabolism of stress-related hormones; they can mitigate abiotic stress by influencing the uptake and transport of ions to maintain ionic homeostasis; and they can mitigate abiotic stress by regulating stress response genes. For example, studies have demonstrated that iron oxide nanoparticles can improve plant growth under saline conditions by modulating ionic balance and enhancing the expression of salt stress-responsive genes.^43^ Similarly, zinc oxide nanoparticles have been observed to mitigate the effects of high salinity in basil plants through the modulation of proline accumulation and antioxidant system activities.^44^ Another key signaling pathway that plants activate primarily in response to abiotic stress is the calcium-dependent protein kinase (CDPK or CPK) pathway,^45,46^ which is currently understudied in terms of nanobased immunomodulation. Strains such as heat; drought; wounding, including cell wall damage by microorganisms or herbivores; and ROS can activate the CDPK pathway, which regulates plant growth, development, and stress response. Essentially, the CDPK pathway is a Ca sensor triggered by a salt imbalance, i.e., by an increase in the concentration of calcium ions in the cytosol. The CDPK pathway causes phosphorylation of downstream target proteins, resulting in the transcriptional regulation of stress-response genes. This, in turn, can improve plant resistance to abiotic stresses, including cell wall damage by herbivores.^46^ In addition, nanoparticle-mediated abiotic stress responses involve MAPK cascades and ROS signaling.^47^

Generally, due to crosstalk, defense-related signal transduction pathways are interconnected in highly complex signaling networks (Figure 2b).^33,48^ The interplay of PTI, ETI, and abiotic stress signaling is not well understood.^14,49^ Based on the physicochemical properties of nanoparticles, depending on the dose, size, shape, and composition, nanoparticles can be expected to interfere upstream with both biotic and abiotic stress defense signaling pathways in plants if they a) damage the cell wall (CDPK and MAPK pathway ↑), b) get stuck in the apoplast (cell walls, root hairs, or stomata) and interfere with evapotranspiration and photosynthesis^5,50^ (CDPK, MAPK pathways ↑), c) generate or scavenge ROS, e.g., by (photo)catalytic processes^51^ (MAPK pathway ↑ or ↓), d) in case of smaller nanoparticles ≪50 nm, if they penetrate the cell membrane, interact with organelles in the cytoplasm, and mechanically stress the cytoskeleton^41^ (MAPK ↑) or electron transport, and e) release toxic molecules or ions.^41^ For example, ROS, which promote the MAPK pathway,^35^ can emerge from damaged chloroplast membranes due to impaired electron transport chains in the photosystems. It should be noted that only H_2_O_2_ can move outside chloroplast membranes. Other ROS have a very short lifetime limiting their movement away from the source. Nanoparticles clogging the apoplast in the root cap, root hairs, or the stomata^5,52^ can prompt the plant to upregulate its photosynthesis to maintain evapotranspiration. This, in turn, can cause moderate ROS generation and likely the subsequent upregulation of the MAPK pathways, which may be associated with the observed salicylic acid response in SiO_2_-NP-exposed *Arabidopsis*.^5^ Much like mechanic stresses caused by larger plant pests such as caterpillars,^53^ nanoparticles might also induce the recognition of damage-associated molecular patterns (DAMPs) such as a degrading cell wall (Figure 2b), mimicking biotic stress, and lead to PTI. For example, zerovalent nano-Fe (*n*ZVI) particles can generate OH^·^ radicals that can degrade pectins.^54^ However, a direct link to a DAMP-associated plant response, such as camalexin biosynthesis or HR-like cell death, remains to be investigated. Overall, the research on nanoenabled immunity modulation of plants is still in its infancy. Future research is needed to examine the behavior of defense signaling in response to nanoparticle exposure.

## 3 Nanoenabled Immunomodulation—Recent Progress

The conclusions in the present section drawn from empirical studies point to hypotheses that will need further validation in the laboratory and field, especially concerning undesired decreases in crop yield. Conventional chemicals that stimulate plant immunity, for example, SAR-inducing and commercially used salicylic acid analog benzothiadiazole,^15^ were often associated with lower biomasses. In addition to this, several other compounds also participate as inducers of SAR including dicarboxylic acid azelaic acid (AzA), glycerol-3-phosphate (G3P), dehydroabietinal (DA) and pipecolic acid (Pip).^55^ Many of these metabolites can be systemically transported through the plant and probably facilitate communication by the primary infected tissue with the distal tissues, which is essential for the activation of SAR.^56^

A first gene expression analysis that included defense- and immunity-related genes by Tumburu et al. from 2015 has demonstrated opposite MAPK modulations (+2-fold vs −2.6-fold) for TiO_2_ and CeO_2_ nanoparticles ∼25 nm in diameter,^57^ which can be explained with the ROS-scavenging properties of CeO_2_^51^ which likely counteracted the MAPK response. Somewhat contradictory, both materials led to a downregulation of known immunity-related genes. For example, CeO_2_ NPs downregulated the gene expression of *ITGB2, TLR6, HLA-DRB3, TIRAP,* and *HLA-A*.^58^ Similarly, previous research shown a significant reduction in complement factor D (Cfd) expression following long-term exposure to TiO_2_ NPs resulted in autoimmune and inflammatory disease states in mice.^59^ Due to the small size of the *Arabidopsis* seedlings investigated, the biomass was not measured. Still, earlier cotyledon emergence and more fully grown leaves showed beneficial effects on the plants for both nanoparticles, with the CeO_2_ nanoparticles performing markedly better.^57^ Other studies have reported increased abscisic acid synthesis growth after exposure to Ag nanoparticles.^60^ This seems to have helped plants with salt stress tolerance^61^ but unsurprisingly inhibited growth. Interpreting the results of Ag nanoparticle exposure is challenging because these nanoparticles release toxic Ag^+^ ions. Thus, it is unclear which part of the plant response has been caused by solid nanoparticles. A similar upregulation of abscisic acid and cell wall reinforcement-related genes was observed in maize under La_2_O_3_ nanoparticle exposure.^62^ Here, the presence of abscisic acid was directly demonstrated by high-performance liquid chromatography. Again, the nanoparticle exposure resulted in growth inhibition. Unfortunately, lanthanum alone can also be toxic to plants in the tested concentration range.^63^ Again, this raises questions about the dissolution of the particles and bioaccessibility of toxic La^3+^ ions. Although to be interpreted with some caution, these results suggest that nanoenabled induction of abscisic acid can be helpful for plant defense, for example, against insect herbivores,^64^ but will most likely result in reduced crop yield. Nanoenabled agricultural strategies focusing on triggering this hormone must consider this caveat.

Ag, SiO_2_, and ZnO nanoparticles modulate plant defenses mediated by ET.^65,66^ For Ag nanoparticles, a downregulation of aminocyclopropane-1-carboxylic acid synthase (ACC synthase, ACS), a key enzyme in ethylene biosynthesis, went along with increased plant health in *Tecomella undulata* (a desert tree) in vitro cultures.^65^ For ZnO nanoparticles, reduced growth of *Arabidopsis* went along with upregulated ET-related genes,^66^ which can be explained by Zn^2+^ toxicity, given the test concentrations >10 mg L^–1^, and the high solubility of ZnO nanoparticles.^67^ Interestingly, SiO_2_ nanoparticles promoted the plants’ growth and chlorophyll contents at all concentrations, induced only a mild upregulation of ET-related genes, and reduced ZnO nanoparticles’ toxicity when used in combination.^66^ Thus, downregulation or moderate ET stimulation by Ag and SiO_2_ nanoparticles seems to be associated with positive outcomes in plant health, which could be helpful under stresses such as flooding or waterlogging.^49^

SA-induced defense responses have the potential to be effective against insects such as aphids^68^ and thrips.^69^ Nanoparticles can affect the concentration of SA, an essential plant hormone for SAR. For example, SiO2,^5^ ZnO,^70^ and Fe_3_O_4_^**3**^ can affect salicylic acid levels. It is also recognized that the metal ions released from nanoparticles due to transformation in soil or plant might also contribute to such modulation, in addition to the nanospecific effects. The effects of ionic Si, Fe, and Zn on gene expression and hormone levels in plants have been well-known. Si has been associated with upregulation of genes involved in stress responses, defense mechanisms, and cell wall reinforcement.^71^ In addition to this, it has also been shown that Si can upregulate the levels of genes associated with oxidative stress.^72^ Fe and Zn deficiency induces the upregulation of genes involved in nutrient uptake and transport mechanisms.^73^ Previous study showed that Si nanoparticles upregulate genes related to stress response, antioxidant defense and photosynthesis more than bulk Si, which is partially due to the discrepancy in the released Si ions.^74^ Fe and Zn nanoparticles have been shown to upregulate genes associated with nutrient uptake and transport.^75^ However, nanoscale Fe and Zn particles exhibited enhanced bioavailability and plant uptake compared to ionic Fe and Zn, leading to differences in gene expression, suggesting that both particles and ions play crucial roles in the immunomodulation.^76^ Some nanoparticles like Fe-based nanoparticles have nanoenzymatic activity to maintain cellular homeostasis by directly scavenging ROS generated by oxidative stress, which is specific to nanoparticles only.^77^ In addition, nanoscale particles may produce longer-lasting and faster hormone responses than ionic.^78^ Due to their smaller size and enhanced stability, nanoparticles may retain their regulatory effects on plant hormone signaling pathways for a longer period of time.

The most stimulating effects of nanomaterials on the immune system repeatedly occurred at low, nontoxic concentrations, possibly related or equivalent to hormesis.^79^ For example, low concentrations of ZnO nanomaterials can induce defense in *A. thaliana* via increased stress hormone levels. Similar observations were made for CuO and Ag particles.^80^ However, higher ZnO nanoparticle concentrations were consistently toxic for the plant.^66,70^ Overall, these findings show the potential for using different nanomaterials at low concentrations to stimulate plant hormones against pests. With proper size tuning, they might also penetrate plant membranes for targeted applications.^81^ However, more research is needed to fully understand how different properties of nanomaterials inside plants can influence hormone stimulation against pests.

## 4 Full Life Cycle Environmental Impact of Using Nanomaterials

While the conventional use of simple chemicals such as methylmalonic acid, N-substituted phthalimides and JA is a well-known method for inducing mild stress in plants,^82,83^ leading to immune response modulation, these methods often lack the precision and targeted action that nanomaterials can offer. Nanomaterials can be engineered to interact specifically with key signaling pathways and molecular targets within the plant’s immune system, offering a level of specificity that conventional chemicals typically cannot achieve. This targeted approach is critical for minimizing unintended effects on the plant and the surrounding environment. Additionally, nanomaterials can provide controlled release mechanisms and reduce the overall quantity of active substances required, potentially decreasing toxicity and environmental impact compared to traditional chemicals. The adaptability of nanomaterials allows for the design of multifunctional agents that can concurrently address various aspects of plant health and stress resilience simultaneously. This contrasts with conventional chemicals, which generally target a narrower range of biological pathways.

When considering the full life cycle environmental impact of different methods to enhance plant immunity, nanomaterials usually require less raw materials for their production, which eases the pressure on the natural resources, comparing with conventional chemicals. Also, the process of making nanomaterials can be more energy-efficient compared to the production of traditional agricultural chemicals due to the less required amount. Physical methods, like controlled drought or adjusting temperatures, are environmentally friendly alternatives. They avoid the use of synthetic substances and generally use a moderate amount of energy. Yet, these physical approaches may cause indirect impacts, such as changes in water usage and effects on the ecosystem, especially when used on a large scale. On the other hand, traditional chemicals, including antibiotics, pesticides, and fungicides, carry significant environmental concerns. Their production processes are often resource-intensive and harmful to the environment. After their application, these chemicals may persist in ecosystems, affecting other organisms and, for example, in the case of antibiotics, contributing to resistance development. Disposing of these chemicals and their containers can also lead to soil and water pollution. Overall, while nanomaterials and physical stress methods offer promising alternatives to conventional chemicals, it is essential to thoroughly weigh the potential risks and benefits of each approach throughout their entire life cycle. By examining the resource consumption, energy requirements, waste generation, and ecological impacts of these strategies, we can make better-informed decisions for sustainable agriculture practices that improve plant immunity and resilience while reducing environmental harm. Likewise, the life-cycle of nanobased plant immune-modulator including and their production, transport, transformation and bioaccumulation should be elucidated before it can be upscaled and applied.

## 5 Key Avenues to Use Nanoenabled Plant Immunomodulation for Agriculture

Here we have discussed the first available research on nanoenabled immunomodulation. Based on these results, we propose avenues to exploit nanoenabled immunomodulation—nanopriming—for agriculture. However, with all the possible benefits in mind, it is essential to consider the potential effects on nontarget organisms. Therefore, all nanoparticles for potential use in agriculture must be designed responsibly, adhering to safe-by-design principles, most importantly, with nontoxic base materials and short weeks-months half-lives.

1. Principal immunity-related plant signaling pathways that lend themselves to nanomodulation are the upstream MAPK cascades that ROS can amplify. They cause a wide variety of different immunological responses, including plant defense hormones. MAPK pathways are exciting targets to strengthen plants against biotic stress. The CDPK pathway is another interesting upstream cascade that can be triggered by abiotic stresses such as heat, drought, and wounding. ROS are ubiquitously involved in these plant immune responses, can amplify the MAPK pathways, and seem to trigger, when used in moderation, a sustained increase of plant resistance. ROS are particularly interesting in modulating plant immunity because specific nanomaterials can either scavenge ROS such as CeO_2_ or induce ROS such as Ag and CuO.^80^ Photocatalytic nanomaterials that have occasionally proved beneficial for plant health include but are not limited to Ti and Ce-based particles.^9,57,84^ Future research should aim at fine-tuning the nanoparticle doses and designs and understanding the relationship between ROS exposure and different plant signaling pathways.
2. Another promising and related research avenue is to induce strong plant resistance (SAR) by nanopriming in general. Nanomaterials can simulate moderate stress and prompt the plant to take defensive measures and prepare for a faster future reaction. Plant priming induced by other biotic and abiotic factors, including salt stress or pathogen attacks, has already been demonstrated.^27^ Despite the absence of effector-triggered immunity in this process, the resulting induced resistance can be strong, systemic, and sustainable, as has already been demonstrated for MgO and SiO_2_ nanoparticles in plants.^5,6^ However, field trials are needed to validate the scalability and effectiveness of nanopriming under realistic conditions.
3. Advanced nanocomposites, including nanomaterials with enzyme-like properties (nanozymes) and transport systems carrying plant nutrients or hormones, are another exciting research avenue. Nanovesicles are particularly suited for delivering volatile organic compounds, such as signaling molecules.^85^ Other particles mainly expected to act mechanically or as carriers of other ingredients are carbonaceous nanomaterials, including C-dots. Future customized composite nanoagrochemicals might boost plant health by releasing various active ingredients at different plant growth stages. For example, CeO_2_ nanoparticles quench ROS, Zn to promote drought tolerance, S to promote the glutathione system and serve as an antibiotic, Fe and manganese (Mn) to boost photosynthesis, and phosphorus when the plant needs it most in the last third of its growth cycle. The obstacles to overcome with such technologies are complex syntheses, toxic reaction byproducts, and high product costs.
4. Nanoenabled macro- and micronutrients, or nanofertilizers, can be expected to induce plant immunity, such as N (chitosan), P, K, Ca, Mg, B, Mn, Zn, Mo, Cu, Fe, and Si.^5,86^ There are also nanoimmunomodulatory effects based on polysaccharides or secondary metabolites. Here, carefully designed mechanistic studies are needed to compare the plants’ immunological response with the response to the ionic counterparts.
5. Finally, immunomodulatory effects can also be helpful in the absence of stress. For example, photosynthesis can be enhanced (better electron capture and transport, enhanced carbon fixation), earlier flowering can be stimulated, and life cycles can be shortened.^51,87^ Such effects could neutralize some of the damage caused by anticipated increasing crop yield losses due to climate change.^7^

In summary, we have identified several strategies for plant immunomodulation for higher crop yields and less strain on the ecosystem. We must carefully investigate the underlying mechanic and molecular mechanisms, the optimal mode of application, and potential consequences for people and the environment. Also, in the field, plants are exposed to many different stresses, such as heat, drought, and pathogens, simultaneously.^49^ Therefore, more quantitative field studies are needed. If such studies can be realized in the near future, and a multidisciplinary research community works in a concerted effort focused on future scalability, the goal of higher crop yields through nanomaterials is attainable within a few years.
